# Eschar- A Forgotten Focus of Concern

**DOI:** 10.4103/0974-777X.62867

**Published:** 2010

**Authors:** S Senthilkumaran, N Balamurgan, V Karthikeyan, P Thirumalaikolundusubramanian

**Affiliations:** *Department of Accident, Emergency & Critical Care Medicine, Sri Gokulam Hospital & Research Institute, Salem, Tamil Nadu, India*

Sir,

Scrub typhus is an acute febrile illness usually endemic in many parts of Asia including India, caused by a microbe Rickettsia *tsutsugamushi (R. orientalis)* and transmitted to humans by the bite of larval stage of infected trombiculid mites or chiggers. Though transmission occurs throughout the year in tropical areas, in India the peak occurs between October and February.[1] At the site of mite bite, an eschar of 5-20 mm in diameter develops. The occurrence varies from 46 to 92%, and influenced by the complexion of the patient. In this letter we stress the importance of eschar in diagnosing the scrub typhus fever.

**Figure 1:**
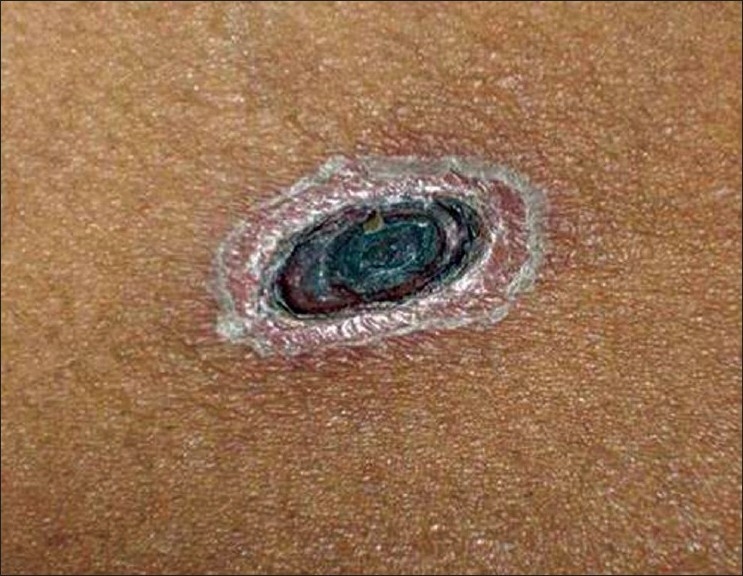
An ulcerated lesion with crust

A 42-year-old male farmer was admitted with the history of fever and generalized body ache of three days duration. On examination he was conscious and febrile. His vitals were stable. He had icterus, axillary lymphadenopathy and a punched out ulcer with blackened scab, the eschar, below the right clavicle [Figure 1]. The vicinity of the eschar was surrounded by erythema. His Weil-Felix test was positive with OXK 320. Biochemical investigations revealed deranged liver and renal function tests. Fever work up for malaria, leptospirosis and enteric was negative. His blood and urine culture was sterile. He was diagnosed as a case of scrub typhus. He was managed with high flow oxygen, doxycycline, azithromycin and supportive care. He responded well to treatment.

The eschar begins as a small papule, then enlarges, undergoes central necrosis, and eventually acquires a blackened crust with an erythematous halo that resembles a cigarette burn. Necrotic eschar at the inoculating site of the mite is pathognomic of scrub typhus as shown in the picture. Eschar heralds the onset of symptoms invariably as observed in this case and usually occurs at sites where skin surfaces meet or clothes bind, such as the axilla, groin, neck, waist, and at in uncommon sites like wrist joint, elbow joint and inguinal area.[2] Although eschars are important in the diagnosis of scrub typhus, the lesions are painless and without any itching sensation in most cases, causing the infection goes undetected. Dong-min Kim *et al*.[3] have suggested assessing the presence of draining lymph node enlargement which could be an important adjuvant method for the diagnosis.

Physicians sometimes encounter eschar-like crust lesions in clinical practice which is similar to a scab formed after trauma, and its size may be very small, which also delays recognition of eschar in many cases. Under such circumstances it is essential to search for eschar carefully on entire body, and suspect scrub typhus as prompt treatment is necessary to decrease mortality in this disease. Lee *et al*.[4] used PCR method to arrive at early diagnosis using eschar material and has differentiated other eschar-like crusted lesions.
